# Combined myocardial stress perfusion imaging and myocardial stress tagging for detection of coronary artery disease at 3 Tesla

**DOI:** 10.1186/1532-429X-10-59

**Published:** 2008-12-18

**Authors:** Daniel Thomas, Katharina Strach, Carsten Meyer, Claas P Naehle, Sebastian Schaare, Sven Wasmann, Hans H Schild, Torsten Sommer

**Affiliations:** 1 Department of Radiology, University of Bonn, Bonn, Germany; 2 Department of Internal Medicine II, University of Bonn, Bonn, Germany

## Abstract

**Background:**

Adenosine stress perfusion is very sensitive for detection of coronary artery disease (CAD), and yields good specificity. Standard adenosine cine imaging lacks high sensitivity, but is very specific. Myocardial tagging improves detection of wall motion abnormalities (WMAs). Perfusion and tagging cardiovascular magnetic resonance (CMR) both benefit from high field imaging (improved contrast to noise ratio and tag persistence). We investigated the diagnostic impact of a combined stress perfusion-tagging protocol for detection of CAD at 3 Tesla.

**Methods:**

Stress perfusion and tagging images were acquired in 3 identical short axis locations (slice thickness 8 mm, FOV 320–380 mm, matrix 256^2^). A positive finding at coronary angiography was defined as stenosis or flow limiting restenosis > 50% in native and graft vessels. A true positive CMR – finding was defined as ≥ 1 perfusion deficit or new WMA during adenosine-stress in angiographically corresponding regions.

**Results:**

We included 60 patients (males: 41, females: 19; 21 suspected, 39 known CAD). Myocardial tagging extended stress imaging by 1.5–3 min and was well tolerated by all patients. Sensitivity and specificity for detection of significant CAD by adenosine stress perfusion were 0.93 and 0.84, respectively. The sensitivity of adenosine stress tagging was less (0.64), while the specificity was very high (1.0). The combination of both stress perfusion and stress tagging did not increase sensitivity.

**Conclusion:**

The combined adenosine stress perfusion-tagging protocol delivers high sensitivity and specificity for detection of significant CAD. While the sensitivity of adenosine stress tagging is poor compared to perfusion imaging, its specificity is very high. This technique should thus prove useful in cases of inconclusive perfusion studies to help avoid false positive results.

## Background

Myocardial stress perfusion imaging is a clinically widely used cardiovascular magnetic resonance (CMR) technique for non invasive detection of significant coronary artery disease (CAD) [1-4]. A number of studies could demonstrate that adenosine stress testing can safely be performed in the CMR environment. While adenosine stress perfusion imaging is very sensitive for detection of coronary artery disease, increasing its specificity may be desirable. In comparison, adenosine stress cine imaging using conventional SSFP sequences or echocardiograpy has been shown to be very specific for detection of CAD, however the sensitivity is very low[3,5,6]. However, it could be demonstrated, that the sensitivity for detection of wall motion abnormalities can be improved by the use of myocardial tagging techniques in comparison to cine imaging alone [7-9].

Both, myocardial perfusion imaging as well as myocardial tagging techniques benefit from imaging at high field strength (i.e. 3 Tesla) [10-13]. The increased contrast to noise ratio (CNR) as well as the increased signal to noise ratio allow for high resolution perfusion imaging and the combination with parallel imaging techniques[10,13]. The long T1 of myocardium at 3 Tesla improves tag definition and tag persistence throughout the cardiac cycle, in comparison to imaging at 1.5 Tesla[12,13].

Thus, the aim of this study was first, to integrate myocardial tagging into a comprehensive adenosine-stress perfusion protocol for detection of coronary artery disease (CAD) at 3 Tesla and second, to investigate the additive value of myocardial tagging in a combined adenosine-stress perfusion-tagging protocol for detection of significant CAD in a mixed patient population (known or suspected CAD).

## Methods

### Patient population

The study protocol was approved by the local Ethics Committee and all patients gave written informed consent. The study population consisted of patients (> 18 years old) suspected of having significant occlusive CAD, who were referred to our MR Department for non invasive adenosine stress testing. Patients were excluded because of contraindications to adenosine medication, such as a history of prior myocardial infarction < 3 days, severe arterial hypertension, asthma or severe obstructive pulmonary disease or AV-block > IIa. All patients discontinued anti-anginal medication ≥ 24 hours before the study and were instructed to refrain from caffeinated beverages or food. Other exclusion criteria were general contraindications to CMR such as severe claustrophobia or metal implants/coils in the brain.

### CMR

All studies were performed on a clinical whole-body 3 Tesla scanner (Achieva, Philips Medical Systems, Best, the Netherlands) equipped with 80 mT/m maximum field gradients and a 200 T/m/sec slew rate using a dedicated 6 element cardiac phased-array coil (3 posterior elements, 3 anterior elements). After acquisition of scout images an ECG gated segmented gradient echo sequence (T1-TFE) was used for myocardial perfusion imaging. Three short-axis sections in the basal, midventricular and apical region of the left ventricle were acquired during each heartbeat. Other sequence parameters were as follows: TR/TE = 2.9/1.33 ms, non-selective 90° saturation pulse, TI = 150 ms, flip angle = 15°, slice thickness = 8 mm, matrix 196 × 150, reconstructed to 256^2^, rectangular field of view of 340 – 380 mm, SENSE factor = 2.5. The tagging sequence was acquired in three identical short axis locations. The scan parameters were: TR/TE = 3.7/22 ms, flip angle = 10°, slice thickness = 8 mm, matrix 256 × 175, reconstructed to 256^2^, rectangular field of view of 320 – 370 mm, SENSE factor = 2.5, 16 cardiac phases per RR-interval. A grid tag pattern with a tag separation of 8 mm was applied. From patient to patient both sequences were scanned in alternating order.

**Figure 1:**
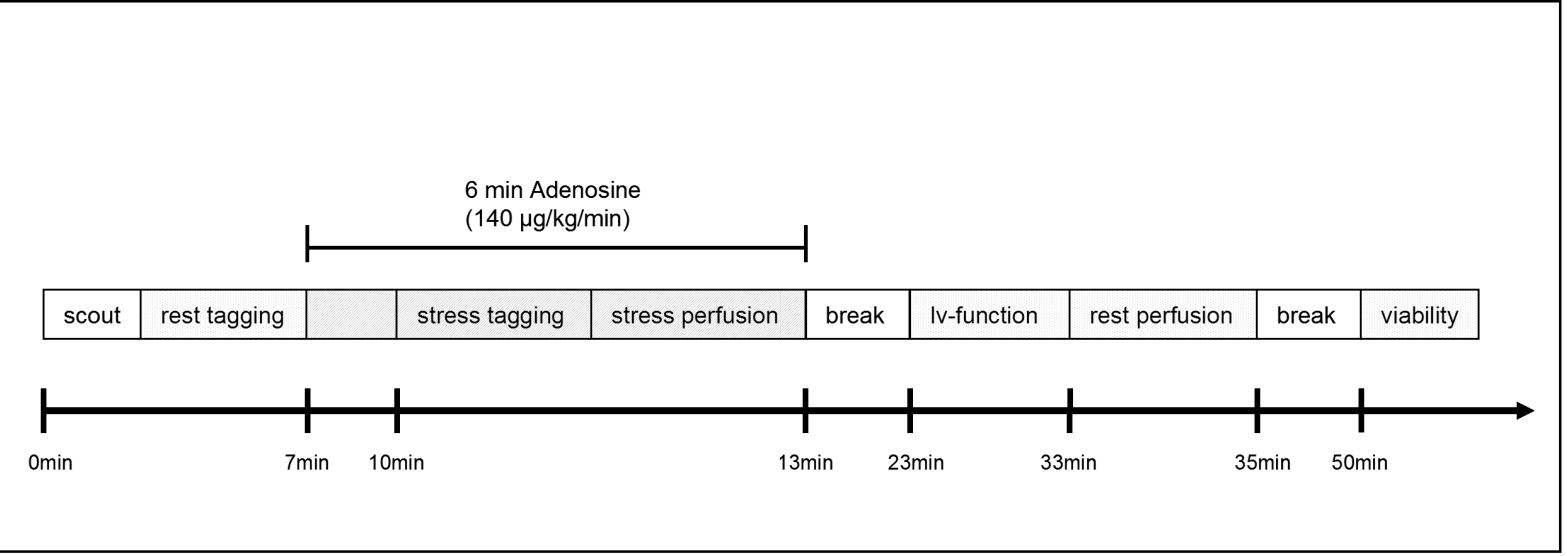
**Time schedule of the imaging protocol**. Stress tagging and stress perfusion were performed in an alternating order on a patient by patient basis.

A standard adenosine infusion protocol was used (Fig. 1): 140 μg adenosine/kg body weight over 6 min, where the stress exams were performed 3 minutes after the beginning of adenosine infusion. The acquisition of perfusion images started simultaneously with the injection of 0.05 mmol/kg gadopentate dimeglumine (Gadovist, Schering, Berlin, Germany) into an antecubital vein at a rate of 5 ml/sec followed by a 20 ml saline flush; images were acquired over 40 sec. Heart rate was monitored continuously and blood pressure was measured at 1 minute intervals during adenosine infusion. Resting studies were performed 30 minutes after stress imaging to allow for adequate clearance of the first bolus of the contrast agent. The acquisition window was adjusted to the patients' heart rate; however, all other scan parameters were kept identical. For the rest scan a slightly higher contrast-agent dose was injected (0.08 mmol/kg). The rest perfusion study was followed by a third injection of contrast agent (up to a total of 0.2 mmol/kg/bw) and late enhancement (viability) imaging for the detection of myocardial infarction was performed approximately 15 min later using a 3D inversion recovery gradient echo sequence.

### Image analysis

All scans were analyzed qualitatively by two experienced readers (D.T., 7 years of CMR experience, T.S., 12 years of CMR experience) through consensus reading. Readers were blinded to the results of invasive coronary angiography. Tagging and Perfusion studies were read on different days and the readers were blinded to the results of either imaging modality. For analysis all slices and segments were assigned to a perfusion territory following AHA guidelines. Rest and Stress perfusion images were displayed side by side and segments were classified as pathologic if they displayed a stress induced perfusion deficit (subendocardial dark rims were interpreted as dark rim artefacts, when already present in the rest perfusion study). Accordingly rest and stress tagging studies were assessed for stress induced wall motion deficits.

A true positive CMR finding was defined as one or more perfusion deficits or new WMA (hypokinesia, akinesia or dyskinesia) during adenosine-stress in an angiographically corresponding region. For comparison of angiographic and CMR data, the type of coronary artery supply (left dominant, right dominant, or co-dominant distribution) was determined from conventional coronary angiography and the coronary artery supply to each segment was assessed according to the AHA criteria[14]. Bypass vessels were assessed according to the respective target vessel territory. Segments 1, 2, 7, 8, 13 and 14 were assigned to the left anterior descending (LAD), segments 3, 9 and 15 to the right coronary artery (RCA) and segments 11 and 16 to the left circumflex artery (LCX). Depending on the type of coronary artery supply, segments 6 and 12 were assigned to the LAD or LCX and segments 4 and 10 to the RCA or LCX. CMR diagnosis of stress-induced ischemia was defined true positive if the involved myocardial segment matched the presumed vascular territory of a significantly diseased coronary artery or the respective diseased bypass vessel at quantitative coronary angiography. All infarcted segments as defined by CMR late enhancement (LGE) were excluded from the analysis, independent of the degree of infarct transmurality.

### Visual assessment of CMR image quality

CMR image quality (rest/stress perfusion and tagging study) was assessed by the two readers based on a four point grading scale. Perfusion images were graded with respect to homogeneity of myocardial enhancement and blurring of epi- and endocardial borders (perfusion study), whereas tagged images were graded with respect to tag definition and tag fading. [5: excellent (no artefacts, good delineation of the endo-/epicardial border, homogenous enhancement; very good tag definition, no significant tag fading throughout the cardiac cycle); 4: slightly impaired (very little artefacts, very little signal inhomogeneities, very little blurring; quite good tag definition, very little tag fading throughout the cardiac cycle); 3: moderately impaired (some artefacts, some signal inhomogeneities, some blurring; some impairment of tag definition, some tag fading throughout the cardiac cycle); 2: severely impaired, (severe artefacts, severe signal inhomogeneity, severe blurring; severely impaired tag definition, significant tag fading throughout the cardiac cycle); 1: non-diagnostic image quality].

### Invasive coronary angiography

Coronary angiography was performed within three weeks after the CMR exam. Invasive coronary angiography was performed in multiple projections using standard techniques. Quantitative angiographic analysis of the studies was performed by an interventional cardiologist according to a standard algorithm. Digital cineangiograms were evaluated using a calibrated off-line analysis package (Cardiovascular Angiography Analysis System mark II, or CAAS II; Pie Medical Imaging, Maastricht, the Netherlands). A clinically significant stenosis was defined as greater than 50% of the vessel diameter in the main coronary arteries, their first order branches or bypass vessels. The reader was blinded to the CMR data.

### Statistical analysis

Analysis was performed on a patient by patient basis as well as a vessel by vessel basis using standard analytical software (MS Excel 2003, Microsoft Corporation, USA). Continuous variables are expressed as mean ± standard deviation. Sensitivity, specificity, accuracy, and predictive values (positive and negative) were calculated according to standard definitions.

## Results

In 60 out of 117 Patients who were referred for adenosine stress testing during the study period, catheter correlation could be obtained. In those 60 patients prevalence of significant CAD was 47%. All of the 60 patients successfully completed the combined adenosine perfusion and tagging protocol. Only minor side effects occurred 2–4 minutes after the onset of adenosine infusion, such as angina (n = 29), dyspnoea (n = 26) headache (n = 11), nausea (n = 13). A second grade AV-Block developed in one patient upon completion of the study and resolved spontaneously after cessation of the adenosine infusion. Detailed demographic patient characteristics are given in table 1.

**Table 1:** Demographic patient data as well as study associated commorbidities.

	**Suspected CAD**	**Known CAD**
**Number of patients**	21	39

**Gender (male/female)**	13/8	28/11

**Age**	54 ± 14	63 ± 12

**Risk factors**		
Hypertension	12 (57%)	32 (82%)
Hypercholesteremia	11 (52%)	33 (85%)
Diabetes	2 (10%)	12 (31%)
Smoking	6 (29%)	17 (44%)
Overweight	6 (29%)	22 (57%)

**Symptoms**		
Typical Angina	11 (52%)	22 (57%)
Atypical Angina	4 (19%)	6 (15%)
Dyspnea	2 (10%)	20 (51%)

**No. of diseased vessels**		
1-VD	2 (10%)	10 (27%)
2-VD	3 (14%)	10 (27%)
3-VD	1 (5%)	19 (49%)
Myocardial Infarction	4	24
LVEF %	64 ± 5	59 ± 8
Bypass/Stent	n.a.	10/26

1 – VD = one vessel disease. LVEF = left ventricular ejection fraction.

### Diagnostic performance

**Figure 2:**
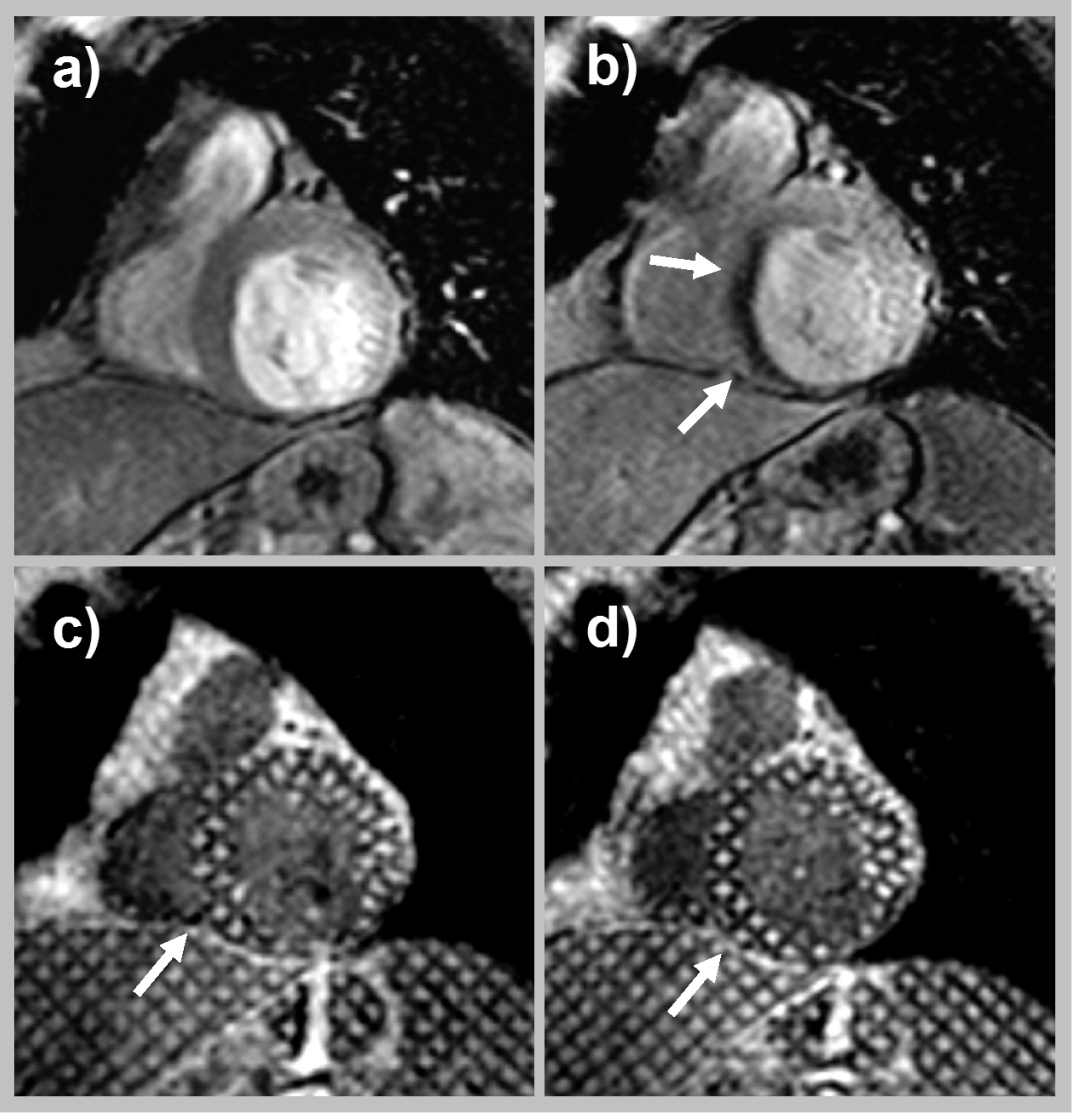
**Images of a 69 year old female with old myocardial infarction and associated thinning of the inferior wall**. The patient was referred because of exertional dyspnea. In comparison to the resting perfusion study (a) the stress perfusion study revealed a near transmural (~75%) perfusion deficit in the septal wall (arrows in b). While demonstrating normal contraction under resting conditions (c) a new wall motion abnormality developed under adenosine stress (arrow in d). Invasive coronary angiography revealed a high grade stenosis of the middle segment of the LAD.

The overall diagnostic performance of adenosine stress perfusion as well as stress tagging for detection of significant CAD is shown in table 2. While stress perfusion imaging revealed the highest values for sensitivity (0.93 vs. 0.64) and a better negative predictive value (0.93 vs. 0.76), stress tagging had a higher specificity (1.00 vs. 0.84). Overall, the accuracy of stress tagging was higher (0.85 vs. 0.83, results are summarized in table 2). The combination of both adenosine stress perfusion as well as tagging did not improve the overall sensitivity, and specificity in comparison to stress perfusion alone. A typical example of a stress induced perfusion deficit with corresponding wall motion abnormality is given in figure 2.

**Table 2:** Overall results of the study based on a patient by patient analysis.

	CMR perfusion	CMR tagging
Sensitivity (%)	0.93	0.64
Specificity (%)	0.84	1
Accuracy (%)	0.88	0.83
Positive predictive value (%)	0.84	1
Negative predictive value (%)	0.93	0.76

The vessel to vessel analysis yielded lowest sensitivities for detection of significant CAD in the LAD territory for both the stress perfusion (sens. 0.69, spec. 0.94) as well as the stress tagging study (sens. 0.46, spec. 1.00). In comparison, sensitivity and specificity for detection of significant CAD was better in the RCA and CX territories using both imaging sequences (RCA: perfusion: sens. 0.92, spec. 0.96, tagging: sens. 0.75, spec. 1.00; CX: perfusion: sens. 0.94, spec. 0.98, tagging: sens. 0.63, spec. 1.00).

Sensitivity for detection of significant CAD was better in patients with suspected CAD than known CAD for the perfusion study (sens. 1.00, spec. 0.81 vs. sens. 0.93, spec. 0.95), while tagging was more sensitive in patients with known CAD (sens. 0.65, spec. 1.00 vs. sens. 0.6, spec. 0.95).

In the group of patients (n = 21) with suspected coronary artery we found four areas of infarct related delayed enhancement in two patients. However, both patients were diagnosed with significant CAD based on an abnormal perfusion scan. Thus, a positive finding of delayed enhancement did not increase sensitivity or specificity in those patients.

### Image quality

There were no significant differences in image quality for both sequences (p > 0.05, perfusion: 3.8 ± 0.78, tagging: 3.8 ± 0.86). Overall no study was graded non-diagnostic. The image quality of 3 perfusion studies in comparison to 6 tagging studies was considered severely impaired.

### Review of false negative and false positive cases

Critical review of the two false negative perfusion studies (patients) revealed that one of the patients had multi-vessel disease, while the other patient had only moderate coronary artery stenosis of 60%. Both false negative perfusion studies were missed by myocardial tagging as well.

There were a total of five false positive perfusion studies. Apparent stress induced perfusion deficits were not related to myocardial infarction and there was no perfusion deficit that could be ascribed to micro-vessel-disease[15]. Missed cases were not related to non diagnostic image quality (all scores > 3).

**Figure 3:**
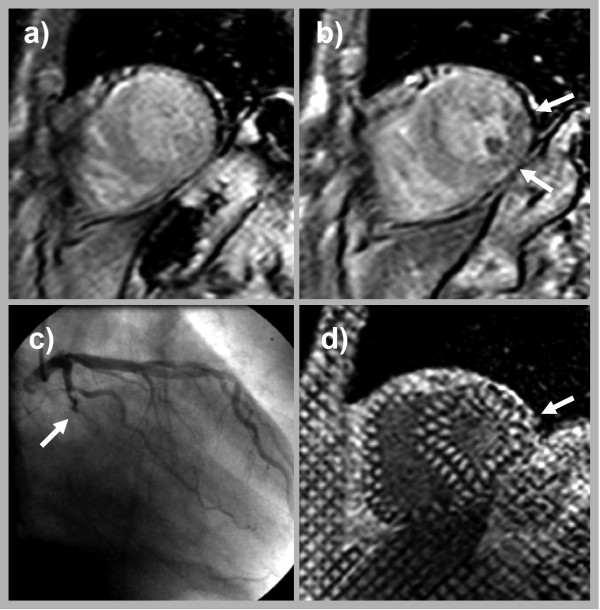
**The stress perfusion study of 67 year old male with recurrent chest pain**. Shows a stress induced transmural perfusion deficit of the inferior and inferolateral wall (arrow in b), consistent with an occluded posterolateral branch (arrow in c). However, stress tagging did not reveal a corresponding wall motion abnormality (arrow in d).

A total of 10 stress tagging studies were false negative. Two of these were missed by stress perfusion as well. In five of the other eight patients hypoperfusion was subendocardial only (5/8) and was limited to one to three segments only (4/8). Perfusion deficits in the other cases (3/8) extended over 2–4 segments. Figure 3 shows a case of a transmural perfusion deficit limited to 2 segments (inferior and inferolateral), which was missed by tagging.

## Discussion

This is the first clinical study implementing myocardial stress tagging into a combined adenosine stress perfusion and tagging CMR protocol at 3 Tesla to screen for coronary artery disease in an unselected patient population. The protocol for detection of significant CAD yielded good values for sensitivity and specificity. While the sensitivity of adenosine stress tagging alone was rather poor compared to perfusion imaging, its specificity was very high.

### CMR stress perfusion

Adenosine stress perfusion imaging has been established as a valuable tool in the detection of significant obstructive CAD[1-4,16]. The vast majority of previous clinical studies using Adenosine stress perfusion imaging have been conducted on 1.5 Tesla clinical CMR scanners, yielding a sensitivity and specificity for detection of CAD in the range of 88–91% and 83–94%, respectively [1-4]. However, CMR-perfusion studies may be hampered by limited spatial or temporal resolution and dark rim artefacts mimicking or obscuring true perfusion deficits[17,18]. Preliminary studies in healthy volunteers and patients suggest an advantage of performing myocardial perfusion imaging at higher field strength[10,11,19]. This advantage is mainly based on the increased SNR and CNR that come with imaging at high field strength. In turn increased SNR and CNR allow for combination with high parallel imaging factors allowing for faster and higher spatial resolution imaging, resulting in an increase of overall image quality as well as reduced dark rim artefacts. The advantages of perfusion imaging at 3T that could be demonstrated in healthy volunteers [10,11] translate into clinical practice in that we observed a very good overall image quality with very little artefacts in our study.

Only recently a study employing a similar scanning approach (imaging sequence and contrast agent dose) has been published comparing diagnostic accuracy of adenosine stress imaging at 3T vs. 1.5T in the same patients[19]. In this cited study myocardial perfusion imaging at 3 Tesla was superior to imaging at 1.5T with regards to SNR and for detection of single vessel and multi-vessel CAD (overall accuracy 90% at 3T vs. 82% at 1.5T). The results of our study clearly support the findings of the cited study. Better spatial resolution and increased SNR as well as CNR as well as a reduction of dark rim artefacts are all in favour of image acquisition and clinical interpretation of perfusion studies at 3T[10,11]. In comparison to other clinical studies performed at 1.5T and first studies at 3 Tesla the sensitivity and specificity in our study for detection of significant occlusive CAD compares favourably well. Contrary to others sensitivity for detection of significant CAD in the LAD-territory was less compared to the CX and RCA-territory. Review of the missed cases though, revealed that all were cases of multi-vessel disease or moderate stenoses.

Among patients with bypasses there was no case of missed significant CAD. However, overall sensitivity in patients with known CAD was less compared to patients with suspected CAD. This may be due to the higher prevalence of significant CAD in general and more specifically of multi vessel disease in this patient population. Overall, sensitivity and specificity of this study were very good in comparison to other studies in unselected patients [5,19] as well as highly selected patients [1].

The results of stress perfusion imaging may further be improved by quantitative or semi quantitative analysis of the data, which will benefit from increased SNR and better spatial resolution as well. We performed a visual analysis in this study, given that this is currently the most widely used approach in clinical practice. Further studies are warranted to determine the optimal dose for first pass perfusion imaging at high field as has been done for imaging at 1.5T. Also, further improvements in shimming technology may allow the use of alternative imaging techniques for myocardial perfusion imaging at 3T (e.g. turbo-FLASH-EPI-readout, or SSFP), who's value needs yet to be determined in clinical studies.

### CMR tagging

Myocardial tagging superimposes a grid pattern on the underlying myocardium. Assessment of tag deformation throughout the cardiac cycle allows not only for evaluation of radial thickening, but also circumferential shortening of the myocardium[9]. Like myocardial perfusion imaging CMR tagging has been shown to benefit from imaging at high field strength[12,13]. This is mostly because of increased tag persistence, which is due to prolonged T1 relaxation times at higher field strength. Together with increased SNR at higher field strength the overall tag definition (tag-myocardium-contrast) is improved. Our clinical study further corroborates those findings; image quality was good to excellent in the majority of patients and in no case graded as non diagnostic.

Previous studies utilizing standard 2D-Echocardiography for detection of inducible wall motion abnormalities (limited to assessment of wall thickening and WMAs) during adenosine or dipyridamole infusion revealed mixed results for sensitivity and specificity for detection of significant occlusive CAD[6,20]. Although it has been shown, that CMR is superior to echo for detection of inducible wall motion abnormalities in high dose dobutamine studies, sensitivity and specificity for detection of inducible WMA under adenosine stress are comparable, when a standard cine gradient-echo imaging sequence is being used [3,5]. However, detection of wall motion abnormalities during stress exams with high dose dobutamine can be improved by myocardial tagging techniques in comparison to standard cine imaging[9]. While detection of WMA by cine imaging alone mostly depends on the assessment of wall thickening and atypical wall motion, tagging reveals information about the contractile behaviour of myocardium in the radial and the circumferential direction, thus adding one dimension to image analysis. Comparable to others we found that the sensitivity of adenosine stress cine imaging is rather low compared to perfusion imaging. However, the overall sensitivity of adenosine myocardial stress tagging appears to be improved to the data reported in a previous CMR-study (64% vs. 40%) employing a standard cine imaging approach for detection of WMAs [5]. Review of the false negative tagging studies revealed that they were associated with perfusion deficits limited to the subendocardium, as well as low grade stenosis. And there is a known relation between the extent of the perfusion deficit and detection of WMAs by adenosine cine imaging, which can be explained by the fact that in the ischemic cascade a perfusion deficit appears before WMAs develop[5].

A quantitative strain analysis was not performed in this study, although a quantitative approach may even increase the accuracy of stress tagging. Even though recent developments in image analysis tools for tagged images have tremendously decreased the time effort for a quantitative analysis[21], it has been our experience that post processing of tagged images is currently still too time-consuming to be implemented in a clinical routine protocol.

There was no case of a positive tagging study and a negative perfusion exam; nevertheless we deem tagging a helpful diagnostic tool in clinical practice, which comes at the cost of three additional breath-holds only and does not significantly prolong the adenosine stress time. A combined stress protocol does extend the diagnostic algorithm in that in case of significantly impaired image quality (severe artefacts) of the perfusion scan the observation of a new WMA by tagging may allow the diagnosis of significant obstructive CAD.

## Conclusion

The implementation of a combined adenosine stress perfusion and tagging protocol at 3 Tesla is feasible and well tolerated by patients. The application of the combined protocol to a mixed patient population with known or suspected CAD delivers good sensitivity and specificity for detection of significant obstructive CAD. Although the sensitivity for detection of CAD by adenosine stress tagging is lower compared to stress perfusion, stress tagging delivers much higher sensitivity for detection of significant obstructive CAD, than previously reported data for standard cine imaging. Thus, stress tagging may be a useful tool in cases of inconclusive perfusion studies (including studies with severe artefacts), to help detect significant CAD or avoid false positive results.

## Competing interests

The authors declare that they have no competing interests.

## Authors' contributions

DT was responsible for design of the study, participated in patient recruitment, MRI study reading, data analysis and manuscript preparation. KS participated in study design, patient recruitment and patient examination. CM contributed to the development of study/scanner protocol, patient examination and statistical analysis. CN participated in patient examination, data analysis and manuscript drafting. SS participated in patient recruitment, patient examination and data analysis. SW participated in the interpretation of clinical data and interpretation of overall data analysis. HS made substantial contributions to the conception and design of the study. TS was involved in manuscript drafting, MRI study reading and critical revision for intellectual content.
